# Correlation between the serum FABP4, ANGPTL3, and ANGPTL4 levels and coronary artery disease

**DOI:** 10.1002/clc.24246

**Published:** 2024-02-29

**Authors:** Zhuoyan Zhao, Ying Fu, Huan Lian, Yixiang Liu, Jingyi Liu, Lixian Sun, Ying Zhang

**Affiliations:** ^1^ Department of Cardiology The Affiliated Hospital of Chengde Medical University Chengde China; ^2^ Hebei Key Laboratory of Panvascular Diseases Chengde China

**Keywords:** ANGPTL3, ANGPTL4, coronary artery disease, FABP4

## Abstract

**Background:**

Lipid metabolism related factors, such as angiopoietin‐like protein 3 (ANGPTL3), angiopoietin‐like 4 (ANGPTL4), fatty acid‐binding protein 4 (FABP4) are newly discovered factors that can affect coronary artery disease (CAD). In this study, we aimed to investigate the relationship between CAD and these lipid metabolism factors.

**Hypothesis:**

ANGPTL3, ANGPTL4, and FABP4 may provide a new method for the control of CAD risk factors and the prevention and treatment of CAD.

**Methods:**

We enrolled 284 consecutive inpatients with suspected CAD and divided them into CAD and non‐CAD groups based on the coronary angiography results. Serum ANGPTL3, ANGPTL4, FABP4, and tumor necrosis factor‐α (TNF‐α) levels were estimated using the enzyme‐linked immunosorbent assay. Multivariate logistic regression was used to assess the risk factors for CAD. The receiver operating characteristic curve was used to determine the cutoff and diagnostic values.

**Results:**

The serum TNF‐α, FABP4, ANGPTL3, and ANGPTL4 values showed a significant difference between the CAD and non‐CAD groups (*p* < .05). After adjusting for confounding factors, the FABP4, ANGPTL3, and ANGPTL4 levels were independently associated with CAD (*p* < .05). The ANGPTL3 expression level was an independent risk factor for CAD in patients with hypertension, but not in those without hypertension. The ANGPTL3 > 67.53 ng/mL, ANGPTL4 > 29.95 ng/mL, and FABP4 > 1421.25 ng/L combination had the highest diagnostic value for CAD.

**Conclusion:**

ANGPTL3, ANGPTL4, and FABP4 were identified as independent risk factors for CAD and have valuable clinical implications for the diagnosis and treatment of CAD.

## Graphical abstract

Our research results indicate that the serum levels of FABP4, ANGPTL3, and ANGPTL4 in the CAD group are higher than those in the non‐CAD group, and they were independent risk factors for CAD. We also found an interaction between ANGPTL3 and hypertension.

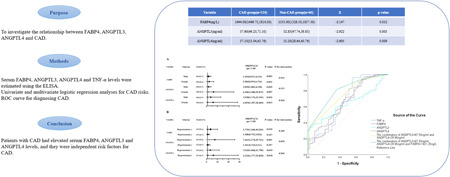

## 1 INTRODUCTION

Coronary artery disease (CAD) is a common cardiovascular disease caused by the formation of atherosclerotic plaques that often lead to coronary artery obstruction. When CAD occurs, blood flow and oxygen levels to the heart are severely reduced, causing angina pectoris and myocardial infarction. According to the American Heart Association, ~19 million deaths worldwide were attributed to cardiovascular disease (CVD) in 2020, which amounted to an increase of 18.7% from 2010.^1^ Although many measures have been applied for the diagnosis and management of CAD, its incidence continues to increase year by year. This indicates that there may be other unknown factors that play an important role in its development and pathogenesis. Identifying and screening individuals with risk factors for heart disease remain crucial for the prevention and treatment of CAD, especially in low‐ and middle‐income countries.

Atherosclerosis (AS) is speculated to be the most common underlying pathology in CAD and is associated with endothelial dysfunction, inflammation, dyslipidemia, foam cell formation, and platelet activation. Mounting evidence demonstrates that some factors related to lipid metabolism play a significant role in the development of AS. Angiopoietin‐like proteins (ANGPTLs) are a family of secreted glycoproteins with a structure similar to angiopoietin and are widely used in the research of obesity,^2^ dyslipidemia,^3^ diabetes,^4^ cancer,^5^, ^6^ cerebral infarction^7^, CVD,^8^ and other diseases. Among the eight proteins in the ANGPTLs, ANGPTL3 is an important regulator of circulating lipid catabolism by inhibiting the activity of lipoprotein lipase (LPL) and endothelial lipase (EL).^9^ ANGPTL4 is also an inhibitor of LPL and can reduce the clearance of AS‐inducing cholesterol. However, ANGPTL4 can delay the progression of atherosclerotic plaques by reducing the inflammatory response to saturated fat, regardless of its effect on lipid levels.^10^ ANGPTL4 participates in lipid metabolism and oxidative stress through different mechanisms and plays different roles in AS. However, the role of ANGPTL4 in CAD patients remains controversial, and more clinical studies are needed. Fatty acid‐binding protein 4 (FABP4) is a member of the lipid chaperone family. FABP4 reportedly participates in AS by influencing oxidative stress and inflammation.^11^ In this study, we aimed to investigate the relationship between the aforementioned factors and their implications in the prevalence of CAD.

## 2 MATERIALS AND METHOD

### 2.1 Study population

A total of 284 patients with suspected CAD were consecutively enrolled in this cross‐sectional study from December 2020 to November 2021 at The Affiliated Hospital of Chengde Medical University. All patients underwent CAG using the Judkins method. The data were independently analyzed by more than two physicians from the cardiology interventional team. According to the CAD diagnostic criteria,^12^ the patients were screened and subsequently classified as follows: (1) non‐CAD group, with one or more coronary stenoses <50% in diameter; and (2) CAD group, with at least one coronary artery stenosis ≥50% (diagnostic of CAD). The CAD group included patients with a vascular diameter measuring >2.00 mm in the left main trunk, left anterior descending artery, left circumflex artery, right coronary artery, or any of their main branches. The exclusion criteria were as follows: coronary artery spasm, secondary angina, myocardial infarction, cardiogenic shock, cardiac arrest, infectious diseases, malignant tumors, severe heart diseases (e.g., aortic dissection and hypertrophic cardiomyopathy), systemic inflammatory disorders, and hepatic and renal dysfunction.

The following patient characteristics were collected: sex, age, body mass index, and histories of hypertension, diabetes mellitus (DM), ischemic stroke, dyslipidemia, family history of CAD and smoking. A single measurement of blood pressure and heart rate were recorded at admission before CAG. Laboratory test results, including those of complete blood count and biochemical tests, were also obtained.

This study was approved by the ethics committee and all patients were informed.

### 2.2 Estimation of inflammation markers

Blood samples from all patients were drawn from the radial artery before CAG. Blood samples were centrifuged at 3000*g* for 10 min, and subsequently stored at −80°C. Serum levels of ANGPTL3, ANGPTL4, FABP4, and tumor necrosis factor‐α (TNF‐α) were measured using an enzyme‐linked immunosorbent assay kit (Jiangsu Meimian Industrial Co., Ltd).

### 2.3 Statistical analyses

Statistical analyses were performed using SPSS (version 26.0; SPSS Inc.) and GraphPad Prism (version 8.0; GraphPad Software Inc.). The Kolmogorov–Smirnov test was used to verify normal distribution of the continuous variables. Data conforming to normal distribution are represented as (*χ* ± s) and measurement data conforming to skewed distribution are represented as M (P25, P75). Quantitative data conforming to normal distribution were compared using Student's *t* test, and data conforming to skewed distribution were analyzed using the Mann–Whitney *U* test. Categorical variables are expressed as frequencies and percentages and were compared using the chi‐squared test. Receiver operating characteristic (ROC) curves were used to calculate the cutoff values of ANGPTL3, ANGPTL4, FABP4, and TNF‐α for diagnosing CAD. Youden's index (sensitivity + specificity − 1) was calculated, and the maximum value corresponded to the optimal cutoff value of these markers. Multivariate logistic regression models were constructed to evaluate the association between ANGPTL3, ANGPTL4, and FABP4 and CAD in the general population and different subgroups. The odds ratio (OR) was determined based on 1 SD increases in serum ANGPTL3, ANGPTL4, and FABP4 levels.

## 3 RESULTS

### 3.1 Patient characteristics

A total of 284 patients were recruited. Their demographic, clinical, and biochemical characteristics are shown in Table 1. The proportion of male patients (67.6% vs. 50.8%, *p* = .013) and those with hypertension (59.4% vs. 41.5%, *p* = .011), DM (39.3% vs. 7.7%, *p* < .001), and dyslipidemia (53.3% vs. 24.2%, *p* < .001) were higher in the CAD group than in the non‐CAD group. Patients with CAD showed significant differences in white blood cell (WBC) count (7.39 × 10^9^/L vs. 6.53 × 10^9^/L, *p* = .001), neutrophil counts (4.94 × 10^9^/L vs. 3.91 × 10^9^/L, *p* < .001), triglycerides (TGs) levels (1.38 mmol/L vs. 1.17 mmol/L, *p* = .009), and high‐density lipoprotein cholesterol (HDL‐C) levels (1.05 mmol/L vs. 1.16 mmol/L, *p* < .001). Moreover, serum TNF‐α (398.15 ng/L vs. 350.65 ng/L, *p* = .001), FABP4 (1644.00 ng/L vs. 1553.00 ng/L, *p* = .032), ANGPTL3 (57.60 ng/mL vs. 52.85 ng/mL, *p* = .003), ANGPTL4 (37.35 ng/mL vs. 35.20 ng/mL, *p* = .009) levels were significantly higher in the CAD group than in the non‐CAD group (Table 1).

**Table 1:** Baseline clinical characteristics of CAD group and non‐CAD group.

Variable	CAD group (*n* = 219)	Non‐CAD group (*n* = 65)	*χ* ^2^/*Z*	*p*
Male	148 (67.6)	33 (50.8)	6.128	.013
Age ≥ 65 years	67 (30.6)	19 (29.2)	0.044	.834
Smoking	124 (56.6)	30 (46.2)	2.212	.137
Hypertension	130 (59.4)	27 (41.5)	6.440	.011
DM	86 (39.3)	5 (7.7)	22.952	<.001
History of stroke	43 (19.6)	8 (12.3)	1.826	.177
Dyslipidemia	115 (53.2)	15 (24.2)	16.327	<.001
Family history of CAD	35 (16.0)	9 (13.8)	0.175	.676
HR	74 (66,82)	68 (64,80)	−1.911	.056
SBP	135 ± 22	135 ± 21	−0.055	.956
DBP	78 (69,88)	79 (72,87)	−0.469	.639
BMI (kg/m^2^)	25.20 ± 3.34	25.54 ± 3.69	0.637	.525
WBC (10^9^/L)	7.39 (5.92,8.96)	6.53 (5.26,7.27)	−3.382	.001
RBC (10^12^/L)	4.48 ± 0.52	4.53 ± 0.44	0.667	.505
NEUT (10^9^/L)	4.94 (3.76,6.64)	3.91 (3.01,5.06)	−3.992	<.001
LYMPH (10^9^/L)	1.47 (1.06,2.03)	1.67 (1.34,1.94)	−1.816	.069
MONO (10^9^/L)	0.45 (0.33,0.65)	0.44 (0.34,0.56)	−0.938	.348
PLT (10^9^/L)	211.00 (172.00,249.00)	220.00 (183.00,268.50)	−1.358	.175
TP (g/L)	69.81 ± 6.48	69.83 ± 5.79	0.018	.986
Alb (g/L)	42.63 (40.05,44.97)	43.30 (40.86,46.35)	−1.420	.156
Urea (mmol/L)	5.63 (4.44,6.78)	5.74 (4.39,6.66)	−0.218	.827
Cr (μmol/L)	64.80 (55.50,75.18)	62.70 (51.10,70.60)	−1.259	.208
UA (μmol/L)	318.97 (259.97,396.77)	304.91 (261.70,374.81)	−0.789	.430
TC (mmol/L)	3.83 (3.03,4.65)	3.77 (3.24,4.26)	−0.428	.668
TG (mmol/L)	1.38 (1.02,2.00)	1.17 (0.78,1.58)	−2.613	.009
LDL‐C (mmol/L)	2.13 (1.54,2.87)	2.17 (1.74,2.57)	−0.066	.947
HDL‐C (mmol/L)	1.05 (0.90,1.26)	1.16 (1.04,1.43)	−3.553	<.001
Hcy (μmol/L)	15.10 (11.68,19.13)	13.30 (11.00,18.10)	−1.270	.204
TBil (μmol/L)	12.25 (9.48,16.63)	11.90 (8.65,14.56)	−1.439	.150
IBil (μmol/L)	9.20 (4.96,13.41)	8.45 (5.89,11.19)	−0.411	.681
TNF‐α (ng/L)	398.15 (339.75,440.65)	350.65 (258.79,439.58)	−3.300	.001
FABP4 (ng/L)	1644.00 (1469.75,1816.00)	1553.00 (1328.50,1837.50)	−2.147	.032
ANGPTL3 (ng/mL)	57.60 (49.25,71.10)	52.85 (47.74,59.85)	−2.922	.003
ANGPTL4 (ng/mL)	37.35 (33.04,43.76)	35.20 (28.64,40.76)	−2.605	.009

Abbreviations: Alb, albumin; ANGPTL3, angiopoietin‐like protein 3; ANGPTL4, angiopoietin‐like protein 4; BMI, body mass index; CAD, coronary artery disease; Cr, creatinine; DBP, diastolic blood pressure; DM, diabetes mellitus; FABP4, fatty acid‐binding protein 4; Hcy, homocysteine; HDL‐C, high‐density lipoprotein cholesterol; HR, heart rate; IBil, indirect bilirubin; LDL‐C, low density lipoprotein cholesterol; LYMPH, absolute value of lymphocyte; MON, absolute value of monocyte; NEUT, absolute value of neutrophil; PLT, platelet count; RBC, red blood cell; SBP, systolic blood pressure; TBil, total bilirubin; TC, total cholesterol; TNF‐α, tumor necrosis factor‐α; TG, triglyceride; TP, total protein; UA, uric acid; WBC, white blood cell.

### 3.2 Univariate and multivariate logistic regression analyses for CAD risks

After adjusting for confounding factors in Model 3, we found that FABP4, ANGPTL3, and ANGPTL4 were independently associated with CAD. The OR of FABP4, ANGPTL3, and ANGPTL4 expression levels per 1 SD increase were 2.404 (95% confidence interval [95% CI]: 1.247, 4.663; *p* = .009), 2.228 (95% CI: 1.172, 4.236; *p* = .015), and 4.842 (95% CI: 1.723, 13.605; *p* = .003), respectively. Therefore, they could be pro‐inflammatory adipokines that affect CAD (Table 2).

**Table 2:** Univariate and multivariate logistic regression analyses of CAD risks according to 1 SD of serum FABP4, ANGPTL3, ANGPTL4 levels.

Variables (per 1 SD increase)	Model 1		Model 2		Model 3	
	OR (95% CI)	*p*	OR (95% CI)	*p*	OR (95% CI)	*p*
FABP4	2.126 (1.274,3.549)	.004	2.347 (1.306,4.219)	.004	2.404 (1.247,4.633)	.009
ANGPTL3	2.445 (1.442,4.144)	.001	2.181 (1.243,3.828)	.007	2.228 (1.172,4.236)	.015
ANGPTL4	3.083 (1.437,6.616)	.004	3.516 (1.478,8.362)	.004	4.842 (1.723,13.605)	.003

*Note*: Model 1: Unadjusted. Model 2: Adjusted for sex, age, BMI, heart rate at admission, blood pressure at admission. Model 3: Adjusted for terms in Model 2 and smoking, dyslipidemia, hypertension, DM, history of stroke, family history of CAD.

Abbreviations: ANGPTL3, angiopoietin‐like protein 3; ANGPTL4, angiopoietin‐like protein 4; BMI, body mass index; CAD, coronary artery disease; CI, confidence interval; DM, diabetes mellitus; FABP4, fatty acid‐binding protein 4; OR, odds ratio.

**Figure 1:**
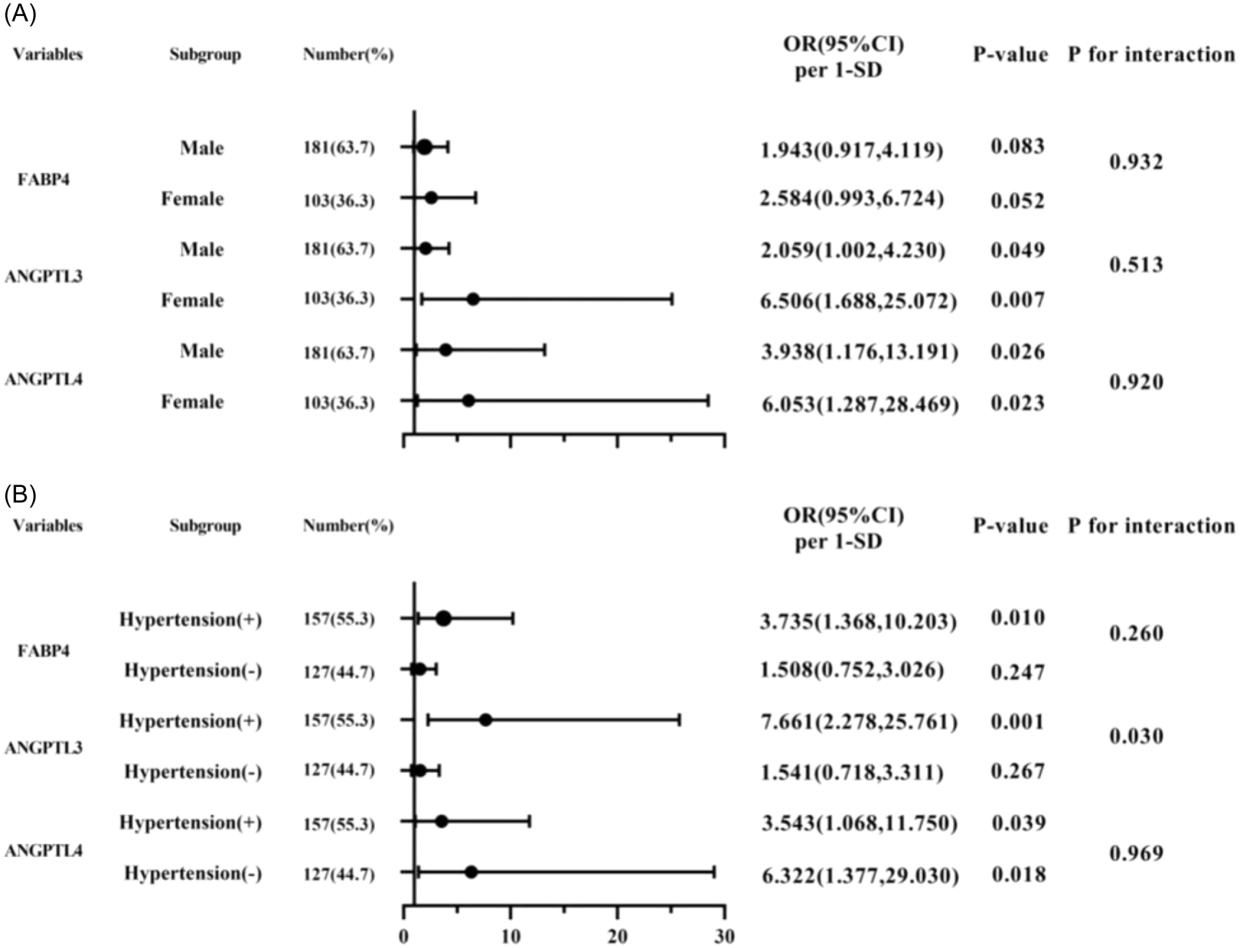
Forest graphs based on subgroup analysis for the effect of fatty acid‐binding protein 4 (FABP4), angiopoietin‐like protein 3 (ANGPTL3), angiopoietin‐like protein 4 (ANGPTL4) in patients with coronary artery disease (CAD). (A) Subgroup analysis according to sex of patients, adjusted for dyslipidemia, hypertension, and diabetes mellitus (DM). (B) Subgroup analysis according to hypertension of patients, adjusted for sex, dyslipidemia and DM. CI, confidence interval; OR, odds ratio.

Subsequently, the independent association between FABP4, ANGPTL3 and ANGPTL4, and CAD was assessed in various subgroups according to sex (male or female) and hypertension (with or without). The FABP4 expression level was an independent risk factor for CAD in patients with hypertension, but not in those without hypertension (OR per 1 SD [95% CI]: 3.735 [1.368, 10.203], *p* = .010 vs. 1.508 [0.752, 3.026], *p* = .247; *p* for interaction = .260). The ANGPTL3 expression level was an independent risk factor for CAD in patients with hypertension, but not in those without hypertension (OR per 1 SD [95% CI]: 7.661 [2.278, 25.761], *p* = .001 vs. 1.541 [0.718, 3.311], *p* = .267; *p* for interaction = 0.030). The results of the subgroup analyses are shown in Figure 1.

### 3.3 ROC curve for diagnosing CAD

**Figure 2:**
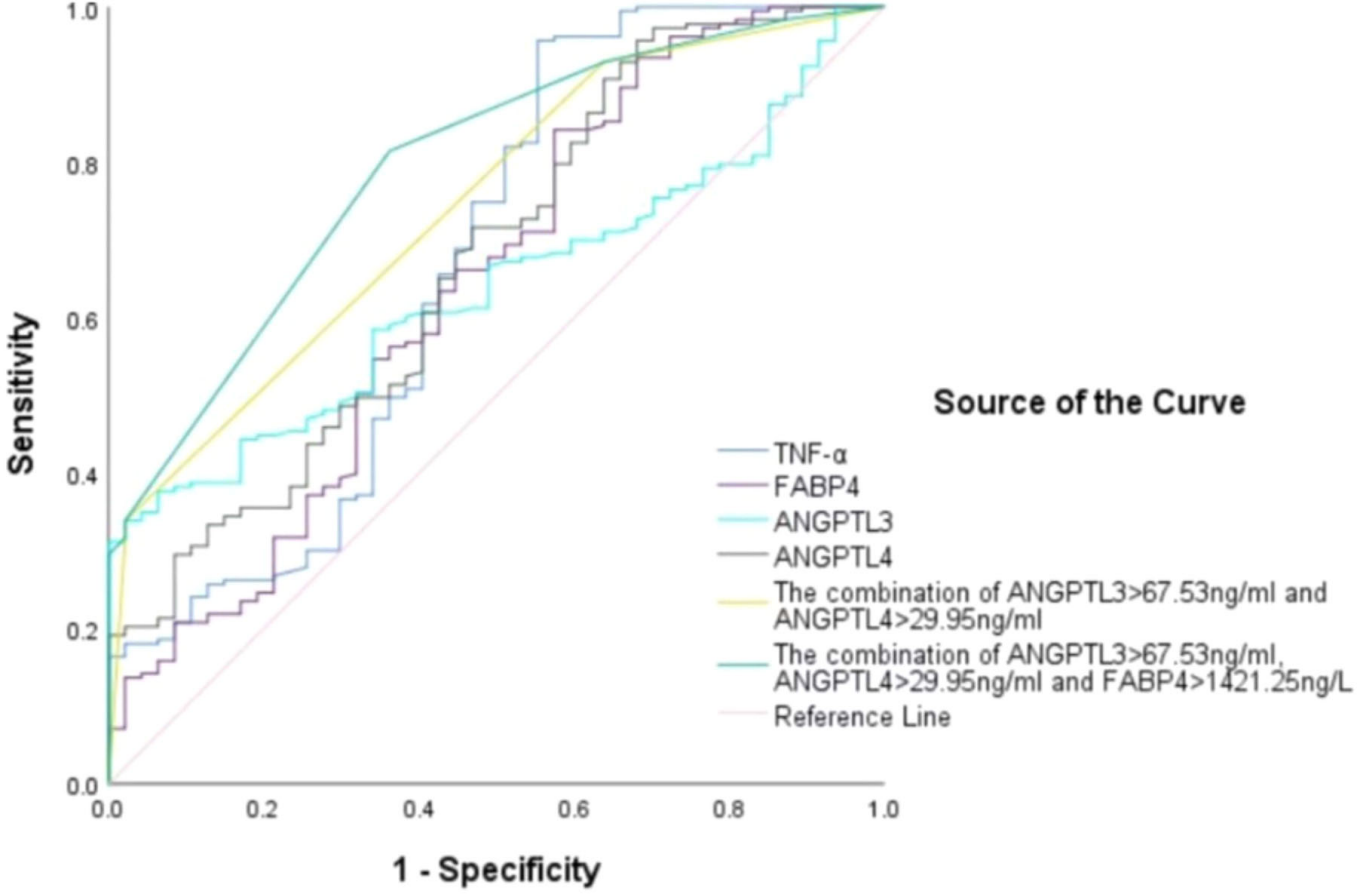
Receiver operating characteristic (ROC) curves of independent and combined diagnosis of coronary artery disease (CAD). ANGPTL3, angiopoietin‐like protein 3; ANGPTL4, angiopoietin‐like protein 3; FABP4, fatty acid‐binding protein 4; TNF‐α, tumor necrosis factor‐α.

Figure 2 and Table 3 present the ROC curve analyses used to determine the optimal cut‐off values of ANGPTL3, ANGPTL4, and FABP4 levels for predicting CAD. The area under the curve (AUC) for TNF‐α, FABP4, ANGPTL3, and ANGPTL4 levels were 0.643 (95% CI: 0.553, 0.733), 0.590 (95% CI: 0.502, 0.678), 0.630 (95% CI: 0.558, 0.702), and 0.608 (95% CI: 0.525, 0.691), respectively. The AUC for the combination of ANGPTL3 > 67.53 ng/mL and ANGPTL4 > 29.95 ng/mL was 0.728 (95% CI: 0.656, 0.800). The AUC of the combination of ANGPTL3 > 67.53 ng/mL, ANGPTL4 > 29.95 ng/mL, and FABP4 > 1421.25 ng/L was 0.774 (95% CI: 0.708, 0.841). The predictive power for CAD improved after combining the values for ANGPTL3, ANGPTL4, and FABPF (all *p* < .05).

**Table 3:** ROC curve analyses for diagnosing CAD.

Variables	AUC	95% CI	*p*	SE (%)	Sp (%)	Cutoff
TNF‐α	0.643	0.553,0.733	.001	93.2	40.4	285.60 ng/L
FABP4	0.590	0.502,0.678	.032	82.1	39.7	1421.25 ng/L
ANGPTL3	0.630	0.558,0.702	.003	30.9	98.1	67.53 ng/ml
ANGPTL4	0.608	0.525,0.691	.009	90.3	31.7	29.95 ng/ml
The combination of ANGPTL3 > 67.53 ng/mL and ANGPTL4 > 29.95 ng/mL	0.728	0.656,0.800	<.001	30.7	98.1	
The combination of ANGPTL3 > 67.53 ng/mL, ANGPTL4 > 29.95 ng/mL, and FABP4 > 1421.25 ng/L	0.774	0.708,0.841	<.001	79.5	60.4	

Abbreviations: ANGPTL3, angiopoietin‐like protein 3; ANGPTL4, angiopoietin‐like protein 3; AUC, area under the curve; CAD, coronary artery disease; FABP4, fatty acid‐binding protein 4; ROC, receiver operating characteristic; TNF‐α, tumor necrosis factor‐α.

## 4 DISCUSSION

In the present study, we investigated the association between FABP4, ANGPTL3, and ANGPTL4, and patients with CAD. The major findings of our study were (1) FABP4, ANGPTL3, and ANGPTL4, as pro‐inflammatory factors, were independent predictors for CAD; (2) the association between ANGPTL3 and CAD was significantly affected by hypertension and there may be interaction between ANGPTL3 and hypertension.

With the development of modern economy and rapid changes in human lifestyle, the incidence of CAD is moving towards a younger population. Additionally, the burden of CAD continues to increase. CAD has a long incubation period and its occurrence and development is relatively slowly. CAD usually develops over several years and decades. In the early stages of CAD, patients and their physicians have plenty of time to arrive at an accurate diagnosis, draft an effective treatment plan, and consider all management options, such as early intervention to prevent or delay the occurrence and development of definitive CAD and its associated complications. Early interventions that address the common risk factors and preventive measures is a key factor in the effective management of CAD. Relying on clinical treatment alone remains inferior to adequate prevention, therefore, early intervention of the most common risk factors is crucial. Management of LDL‐C is a core principle for primary and secondary prevention of atherosclerotic cardiovascular disease. However, despite achieving guideline levels of LDL‐C, patients with AS still have significant residual cardiovascular risk, in part due to mixed hyperlipidemia with elevated fasting and nonfasting TG‐rich lipoproteins.^13^

In previous studies, ANGPTL3 and FABP4 have played a role in promoting the occurrence and development of AS, whereas the role of ANGPTL4 is more complex. FABP4, which belongs to the fat chaperone family. It is mainly regulated by fatty acids, peroxisome proliferator‐activated receptor γ (PPARγ), and insulin. FABP4 is expressed in adipocytes and macrophages, and is a key mediator of local and systemic metabolism and inflammatory processes. In macrophages, FABP4 increases cholesterol ester accumulation and foam cell formation by inhibiting the PPARγ‐hepatic X receptor α‐ATP‐binding box A1 pathway.^14^ FABP4 is also involved in oxidative stress and inflammation. FABP4 reduces cyclooxygenase‐2 and inducible nitric oxide synthase expression in macrophages through IKK‐NF‐κB and JNK‐AP‐1 pathways,^15^ and thus promotes oxidative stress response. FABP4 also regulates the expression of inflammatory cytokines, such as interleukin (IL)‐1β, IL‐6, and TNF‐α, and thus promotes inflammatory necrosis.^15^ FABP4 can also upregulate the expression of adhesion molecules, such as integrin β2, α4, P‐selectin ligand protein, intercellular cell adhesion molecule 1 (ICAM‐1), vascular cell adhesion molecule 1 (VCAM‐1), and P‐selectin, and thus promote ox‐LDL‐induced cell adhesions and AS development.^11^

ANGPTLs, a family of secreted glycoproteins with a structure similar to angiopoietin, are widely expressed in the liver, vascular system, and hematopoietic system and play an important role in inflammation, lipid metabolism, and angiogenesis.^16^ ANGPTL3 is a glycoprotein produced by the liver that inhibits the LPL and EL. The inhibition of ANGLTL3 increases the activity of LPL, which increases the peripheral clearance rate of chylomicrons and very low‐density lipoprotein (VLDL) particles and leads to a decrease in TG.^17^ Increased LPL activity can mediate VLDL hydrolysis, enhance the clearance rate of, and reduce the production of LDL‐C.^18^ In the absence of functional LDLR activity, inhibition of ANGPTL3 may alter the composition of VLDL by increasing EL activity. This results in the rapid removal of residual particles, lipid depletion in the circulation, depletion of the liver LDL‐C precursor pool, and ultimately lowering of the LDL‐C levels.^19^ Some animal studies have also confirmed that targeted inhibition of ANGPTL3 can increase the activity of LPL and EL, and reduce the plasma levels of TG, LDL‐C and HDL‐C.^20^ ANGPTL4 is primarily produced in the liver and adipose tissue in a tissue‐dependent manner after fasting and hypoxia.^21^ In the fasting state, systemic or partial inactivation of ANGPTL4 decreases plasma TG levels, increases LPL activity in adipose tissue, enhances plasma TG clearance, and enhances the uptake of plasma TG‐derived fatty acids into the adipose tissue. In macrophages, the expression of ANGPTL4 is induced by fatty acids, acetylated LDL, oxidized LDL, and natural and synthetic TG emulsions.^22^ ANGPTL4 deficiency in adipose tissue downregulates ICAM‐1, and VCAM‐1 reduces early vascular inflammation in AS‐induced areas.^23^ ANGPTL4 inhibits the aggregation of monocytes/macrophages and the uptake of LDL‐C by macrophages, thus preventing oxidative stress reaction,^24^ reducing foam cell generation, and inhibiting AS occurrence and development.

The serum levels of FABP4, ANGPTL3, and ANGPTL4 were higher in the CAD group than in the non‐CAD group. The FABP4^25^ and ANGPTL3^10^ levels were similar to those in previous studies. However, the ANGPTL4 values differed from those in previous studies.^10^, ^26^ In a study by Yasufumi et al.,^27^ there was no significant difference in plasma ANGPTL4 levels between the CAD and non‐CAD patients. However, ANGPTL4 expression increased in the epicardial adipose tissue of CAD patients. We estimated the serum ANGPTL4 levels in CAD and non‐CAD patients, the serum ANGPTL4 levels in the CAD group was significantly higher than that in the control group. This provides new evidence for the role of ANGPTL4 in CAD.

FABP4, ANGPTL3, and ANGPTL4 are independent risk factors for CAD. We adjusted for confounding factors affecting CAD in the multivariate analysis and found that FABP4, ANGPTL3, and ANGPTL4 were independent risk factors for CAD. In addition to findings of previous studies, FABP4, which is associated with myocardial perfusion abnormalities and left ventricular function, is not only an independent risk factor for CAD,^28^ but also an independent risk factor for heart failure.^29^ Circulating ANGPTL3 levels was also positively correlated with the severity of coronary artery stenosis,^10^ peripheral arterial stiffness^30^ and aortic augmentation index.^31^ Moreover, people with elevated ANGPTL3 levels are at an increased risk of major adverse cardiovascular events.^32^ In the subgroup analysis, the differences in serum FABP4 and ANGPTL3 levels between the CAD and non‐CAD groups in hypertensive patients were more significant than those in nonhypertensive patients. However, the results showed no interaction between FABP4 and hypertensive patients. Moreover, the results showed that ANGPTL3 and hypertension interact with each other in CAD. This suggests that the levels of ANGPTL3 may have a much greater impact on CAD in hypertensive patients than in people without hypertension, which may be due to the fact that patients with hypertension are often complicated with obesity and dyslipidemia,^33^ thus affecting the levels of ANGPTL3, which are mainly secreted by adipose tissue. This is an interesting discovery that can provide a new direction for future research. Our study also showed that elevated serum ANGPTL4 levels were an independent risk factor for CAD. ANGPTL4 deficiency reportedly reduces the risk of CAD by downregulating TG levels,^26^, ^34^ which is consistent with our research findings. Moreover, the serum ANGPTL4 level was negatively correlated with HDL‐C, a protective factor of CAD.^35^ At present, some animal models have confirmed that short‐term treatment with monoclonal antibodies of ANGPTL4 can reduce TG and LDL, thus providing strong evidence for the application of ANGPTL4 targeted therapy in patients with CAD.^26^, ^36^ However, ANGPTL4 also has the effect of inhibiting inflammation. In vitro studies have shown that ANGPTL4 can alleviate apoptosis and oxidative stress of bone marrow mesenchymal stem cells induced by hypoxia and alleviate myocardial damage in rats with myocardial infarction.^37^ The intervention of ANGPTL4 in AS mice can reduce the proliferation of vascular smooth muscle cells and the progression of atherosclerotic plaque.^38^ It has been observed in mice with acute myocardial infarction that the elevated ANGPTL4 level is caused by hypoxia, which has a protective effect on the heart and can even be used to predict the prognosis of the disease.^39^ Since ANGPTL4 also plays a role in inhibiting inflammation, its role and mechanism in CAD requires more basic research. Additionally, more clinical trials are required to determine whether ANGPTL4 antagonists can be applied for the intervention and treatment of risk factors in patients with CAD.

We also evaluated the diagnostic value of FABP4, ANGPTL3, and ANGPTL4 in CAD. Compared with TNF‐α, serum levels of FABP4, ANGPTL3, and ANGPTL4 have lower diagnostic values. We divided them into binary classification variables according to their cut‐off values to evaluate their combined value in the diagnosis of CAD. The AUC of the ANGPTL3 > 67.53 ng/mL and ANGPTL4 > 29.95 ng/mL combination for diagnosing CAD was 0.728, the diagnostic efficiency was improved, and the specificity was high, indicating that the combination of ANGPTL3 > 67.53 ng/mL and ANGPTL4 > 29.95 ng/mL had better value in excluding CAD. The AUC of the ANGPTL3 > 67.53 ng/mL, ANGPTL4 > 29.95 ng/mL, and FABP4 > 1421.25 ng/L combination for diagnosing CAD was 0.774, which was higher than their independent diagnostic values. Our results provide new evidence for ANGPTL3, ANGPTL4, and FABP4 as biomarkers for CAD.

### 4.1 Study limitations

Our study has some limitations. First, the data of our patients were from a single center, which may inherently have a selection bias, limiting the generalizability of our findings to some patients with CAD. In addition, the cutoff values of the factors may vary depending on the patient population, which may not be applicable to patients with other CVDs or CADs in other countries. Second, because this was a cross‐sectional study, we could not determine the causal relationship between lipid metabolism‐related factors and CAD. Third, the non‐CAD group in this study included patients with no coronary artery stenosis and patients with single or multiple vessel lesions <50%, which may underestimate the difference between the CAD and non‐CAD groups.

## 5 CONCLUSION

We found that patients with CAD had elevated serum FABP4, ANGPTL3, and ANGPTL4 levels, and they were independent risk factors for CAD. The AUC of the ANGPTL3 > 67.53 ng/mL, ANGPTL4 > 29.95 ng/mL, and FABP4 > 1421.25 ng/L combination for diagnosing CAD was the highest. Finally, ANGPTL3, ANGPTL4, and FABP4 are expected to be used as novel diagnostic biomarkers and therapeutic targets for CAD in clinical practice.

## AUTHOR CONTRIBUTIONS

Zhuoyan Zhao and Ying Zhang designed the study, and wrote and revised the manuscript. Experimental operation and data statistics were performed by Zhuoyan Zhao, Ying Fu, Huan Lian, and Yixiang Liu. Jingyi Liu and Lixian Sun contributed to data collection. All authors read and approved the final manuscript.

## CONFLICT OF INTEREST STATEMENT

The authors declare no conflict of interest.

## Data Availability

All data generated or analyzed during this study are included in this article. Further enquiries can be directed to the corresponding author upon urgent request and associated need.
